# Editorial: Intergenerational impacts of perinatal mental health

**DOI:** 10.3389/fpsyt.2025.1542112

**Published:** 2025-01-24

**Authors:** Andri Christoforou, Elif Aysimi Duman, Rafael A. Caparros-Gonzalez

**Affiliations:** ^1^ Department of Social and Behavioral Sciences, European University Cyprus, Engomi, Cyprus; ^2^ Department of Molecular Biology and Genetics, Faculty of Engineering and Natural Sciences, Acıbadem University, Istanbul, Türkiye; ^3^ Institute of Natural and Applied Sciences, Acıbadem University, Istanbul, Türkiye; ^4^ Department of Nursing, Faculty of Health Sciences, University of Granada, Granada, Spain; ^5^ Instituto de Investigacion Biosanitaria ibs., University of Granada, Granada, Spain

**Keywords:** perinatal mental disorders, perinatal mental health, intergenerational impact, perinatal depression, perinatal anxiety, infant neurodevelopment, infant gut microbiota, fetal programming hypothesis

The health and wellbeing of women, children, and families can be impacted in multiple and diverse ways by perinatal maternal mental health problems such as depression, anxiety as well as other psychopathological symptomatology. For instance, previous literature has established that maternal perinatal depression may have intergenerational influences, leading to neurodevelopmental, cognitive, behavioral, emotional, and mental health problems for the offspring that can persist or have effects throughout childhood and adolescence (1–5). While the impact of perinatal anxiety has not been examined as extensively (6, 7), there is, nonetheless, evidence that highlights the association of maternal anxiety with poorer offspring social-emotional, cognitive, and language development, with effects often extending in later years (8). Although there is currently a good understanding in the scientific community of several risk factors for the development of these perinatal disorders, new studies such as those included in this Research Topic enrich our understanding of contextual risk factors that subsequently call for targeted prevention and intervention strategies.

Three articles in this Research Topic are from different countries and participant populations. Chen et al., who explored the effects of biological, psychological, and social factors on maternal depressive symptoms during the COVID-19 pandemic in Jiangsu Province of Eastern China, found that the biological factor of parity was associated with depression in late pregnancy, with multiparous women being more likely to develop depression than primiparous women. In addition, women who exhibited anxiety symptoms, insomnia and COVID-19 fear, along with vulnerability in terms of delays in prenatal care, drinking water quality, family function, and social support were more likely to exhibit depressive symptoms. Abebe et al. examined another overlooked issue in the literature, that is, antenatal depression and its predictors among HIV positive women in Sub-Saharan Africa. Through a systematic review and meta-analysis, the authors conclude that the pooled prevalence of antenatal depression among HIV-positive women in Sub-Saharan Africa is 30.6%. Factors significantly associated with an increased risk of antenatal depression in the population under investigation include being unmarried, having a previous history of depression, experiencing intimate partner violence, and experiencing stigma. Worrall et al. examined the association between prematurity and postpartum-specific anxiety, and the relationship between postpartum-specific anxiety and stress in the NICU in the United Kingdom. Gestational age had a significant negative association with parenting competence and the mother–infant relationship, anxieties around infant illness and accidental harm, and about feeding and routine care. Postpartum-specific anxiety was also positively associated with stress in the NICU, that is, environment-specific stressors including sights and sounds, infant’s appearance, and parental role alteration.

Advances in our scientific understanding of the parameters and pathways that affect the aforementioned associations are vital for the development of targeted and effective prevention and intervention strategies. Researchers within the mental health field are increasingly interested in fetal programming mechanisms, originally identified within the Developmental Origins of Health and Disease (DOHaD) framework, to examine how maternal perinatal mental disorders can impact fetal development, predicting child, adolescent, and adult developmental and mental health outcomes (9–11). Such mechanisms could include, for example, hormonal priming effects (e.g. cortisol), changes in the placenta (e.g. exposure to glucocorticoids), and epigenetic mechanisms (e.g. DNA methylation). Under this Research Topic, Zhou et al. examine the association of maternal postpartum depression (PPD) symptoms with infant neurodevelopment and gut microbiota. Their study with 101 mother-infant dyads derived from a triple-blinded randomized controlled trial suggests that maternal PPD symptoms at day 42 postpartum were negatively associated with infant neurodevelopment at 6 months, as shown by lower scores in the Ages and Stages Questionnaire total score as well as in domains of fine motor, problem-solving and personal-social categories. These differences remained statistically significant after adjusting for confounders such as maternal pre-pregnancy BMI, gestational age, mode of delivery, NICU care and infant feeding. The authors suggest that these differences are associated with changes in the infant’s gut microbiota and metabolites (i.e. increase in alpha diversity and reduction in beneficial bacteria, such as *Bifidobacterium*, and decrease in the neurotransmitter N-Acetyl aspartic acid for infants who had been exposed to maternal PPD symptoms). Zhou et al.‘s study is the first study that establishes an association between maternal PPD symptoms and infant neurodevelopment through changes in the composition of infant fecal microbiota and metabolites.

Further research is needed to examine hypotheses of fetal programming through longitudinal designs that would allow the assessment of offspring for extended periods of time (10). Such studies should also ensure the inclusion of diverse populations and countries, taking into consideration the context-specific conditions and needs of women in low- and middle-income countries (12). Besides enhancing our understanding of fetal programming, future studies can pave the way for evidence-based prevention and intervention programs that optimize maternal, child, and family wellbeing across the lifespan.
